# Efficacy and safety of videolaryngoscopes for transesophageal echocardiography probe insertion: A trial sequential meta-analysis

**DOI:** 10.1371/journal.pone.0311234

**Published:** 2024-10-10

**Authors:** Kuo-Chuan Hung, Wei-Ting Wang, Chia-Hung Yu, Jheng-Yan Wu, Chien-Ming Lin, Wei-Cheng Liu, Tso-Chou Lin, I-Wen Chen

**Affiliations:** 1 Department of Anesthesiology, Chi Mei Medical Center, Tainan City, Taiwan; 2 Department of Anesthesiology, E-Da Hospital, I-Shou University, Kaohsiung City, Taiwan; 3 Department of Nutrition, Chi Mei Medical Center, Tainan City, Taiwan; 4 The Department of Occupational Therapy, Shu-Zen junior College of Medicine and Management, Kaohsiung City, Taiwan; 5 Department of Anesthesiology, Tri-Service General Hospital, National Defense Medical Center, Taipei, Taiwan; 6 Department of Anesthesiology, Chi Mei Medical Center, Liouying, Tainan City, Taiwan; Beth Israel Deaconess Medical Center / Harvard Medical School, UNITED STATES OF AMERICA

## Abstract

**Objective:**

This meta-analysis aimed to compare videolaryngoscope (VL)-assisted transesophageal echocardiography (TEE) probe insertion with conventional methods in terms of efficacy and safety.

**Methods:**

Several major databases such as Medline and Embase were systematically searched to identified relevant studies from inception to June 2024. The primary outcome was complication rate, defined as the proportion of patients experiencing complications related to TEE probe insertion. Injuries at specific sites (e.g., posterior hypopharyngeal wall) from both groups were also analyzed. The secondary outcomes included the first-attempt success rate and total insertion time of VL and conventional methods.

**Results:**

Seven trials involving 716 participants were identified. The use of VL was found to significantly reduce the complication rate (risk ratio[RR]:0.28, 95% confidence interval[CI]:0.17–0.46, *P* < 0.00001) and increased the first-attempt success rate [FASR] (RR:1.33, 95%CI: 1.10–1.60, *P* = 0.003) compared with conventional methods. These findings were confirmed by trial sequential analysis. No significant difference was found in the TEE insertion time among the two techniques (mean difference: −2.94s, 95%CI: −10.28–4.4, *P* = 0.43). VL significantly reduced the risk of trauma to the hypopharyngeal wall but showed no significant benefits in other areas (e.g., pyriform sinus). The certainty of evidence was moderate for the complication rate, very low for the FAS rate, and low for the TEE insertion time.

**Conclusion:**

The use of VL for TEE probe insertion is associated with a significantly lower complication rate and higher FAS rate than conventional methods. These findings suggest that VL enhances patient safety and improves the efficiency of TEE probe insertion.

## 1. Introduction

Transesophageal echocardiography (TEE) is a well-established diagnostic and monitoring tool that is widely used in cardiac surgery, intensive care units, and cardiology practice [1–4]. It is also commonly employed in various major surgical interventions, such as lung and liver transplantations [5–7]. Despite its many advantages, TEE probe insertion is associated with complications, such as oropharyngeal injury, esophageal perforation, and gastrointestinal bleeding [8–11]. The most frequently reported complication is trauma to the upper gastrointestinal tract, with incidence rates ranging from 0.2% to 1.4% [8–10,12]. Other injuries include oropharyngeal injuries, such as damage to the uvula, pharynx, or glottis, with incidences ranging from 0.03% to 0.11% [12,13]. Severe complications, such as esophageal or hypopharyngeal perforation, although rare, can be life-threatening and cause medical malpractice claims [14–16]. In anesthetized and intubated patients, TEE probe insertion can be particularly challenging because of the loss of upper-airway muscle tone and the presence of an endotracheal tube [17]. Conventional blind insertion techniques may require multiple attempts, which increases the risk of oropharyngeal trauma and other complications. To address these concerns, various techniques and instruments that facilitate safer TEE probe insertion have been proposed, including the use of soft-tipped esophageal bougie as a guide [18], jaw thrust maneuvers [19], and rigid laryngoscopy [20].

VL has been reported as a promising device for enhancing the safety and efficacy of TEE probe insertion in several case studies [21,22]. By providing a better view of the larynx and esophageal inlet, it may reduce the risk of oropharyngeal injury and facilitate successful probe insertion [23]. Previous randomized controlled trials (RCTs) have evaluated the utility of VL for TEE probe insertion to reduce complication rate and insertion attempts [17,24,25]. However, these trials were limited by relatively small sample sizes, necessitating the aggregation of evidence for clinical application. Although Namekawa et al. [26] previously conducted a meta-analysis evaluating the utility of VL for TEE probe insertion, the inclusion of only three studies in their meta-analysis restricted the applicability of the evidence. To compensate for the knowledge gap, we conducted this systematic review and meta-analysis to compare VL-assisted TEE probe insertion and conventional methods in terms of efficacy and safety. By pooling data from available RCTs, we aimed to conduct a more robust and generalizable assessment of the potential benefits of VL in reducing complications, increasing first-attempt success rates, and minimizing insertion time across diverse patient populations and clinical settings.

## 2. Methods

### 2.1. Protocol registration

This meta-analysis adhered to the RRISMA guidelines (S1 Table). To ensure transparency and reduce the risk of reporting bias, the protocol was registered with the International Prospective Register of Systematic Reviews before the literature search (Registration number CRD42024554896).

### 2.2. Search strategy

The Medline, Google Scholar, Embase, and Cochrane Central Register of Controlled Trials databases were systematically searched to identified RCTs that compared the efficacy and safety of VL-assisted TEE probe insertion with conventional methods. The search strategy involved the use of both Medical Subject Headings terms and free text keywords pertaining to VL and TEE probe insertion. A tailored search strategy for each database included key terms such as “videolaryngoscope,” “video laryngoscope,” “Airtraq,” “Airwayscope,” “King Vision,” “GlideScope,” “C-MAC,” “McGrath,” “Pentax-AWS,” “transesophageal echocardiography,” “TEE,” “TEE probe,” and “TEE probe insertion.” The searches were restricted to peer-reviewed articles from the inception of each database to June, 2024. The reference lists of the related articles were carefully reviewed manually. Table 1 presents the detailed search strategy for one of the databases (i.e., Medline).

**Table 1:** Search strategy for Medline.

1	("transesophageal echocardiography " or "TEE" or "TEE probe insertion" or "Transesophageal ultrasound" or "Transesophageal echo" or "Transesophageal sonography").mp.
2	exp "Echocardiography, Transesophageal"/
3	("Videolaryngoscope" or "video laryngoscope" or "Airtraq " or "Airwayscope" or "King Vision" or "GlideScope" or "C-MAC" or "McGrath" or "Pentax-AWS").mp.
4	("Blind technique" or "Jaw thrust" or "Blind insertion" or "Laryngoscope" or "Macintosh").mp.
5	exp "Laryngoscopes"/
6	(1 or 2) and 3 and (4 or 5)
7	6 and (((randomized controlled trial or controlled clinical trial).pt. or randomi*ed.ab. or placebo.ab. or drug therapy.fs. or randomly.ab. or trial.ab. or groups.ab.) not (exp animals/ not humans.sh.))

### 2.3. Eligibility criteria

The inclusion criteria for the studies were as follows:

- Population (P): adult patients that required TEE probe insertion regardless of the clinical setting (e.g., operating room or intensive care unit)
- Intervention (I): the use of VL for TEE probe insertion (i.e., VL group)
- Control (C): the use of conventional methods, such as blind insertion or direct laryngoscopy, for TEE probe insertion (control group)
- Outcomes (O): studies documenting at least one of the following: success rate of TEE probe insertion, time required for successful insertion, or complications (such as posterior hypopharyngeal wall trauma). Current meta-analysis only included RCTs for analysis.

The exclusion criteria were nonrandomized studies, case reports, case series, editorials, letters, and review articles. Studies that involved pediatric patients or manikins or those that failed to report any of the specified outcomes were excluded. Two reviewers independently screened the titles and abstracts of the retrieved articles to identify studies that met the criteria. The full texts of these articles were then reviewed for final inclusion. Any differences of opinion between the reviewers were settled through discussion.

### 2.4. Outcomes

The primary outcome in current meta-analysis was complication rate, defined as the proportion of patients experiencing complications related to TEE probe insertion, such as oropharyngeal trauma, dental injury, or bleeding. Injuries at specific sites (e.g., posterior hypopharyngeal wall) from both groups were also analyzed. The secondary outcomes included the first-attempt success rate and total insertion time of VL and conventional methods.

### 2.5. Data extraction

Two reviewers independently gathered data from the included studies using a standardized data extraction form, collecting the following information:

study characteristics (first author, country, and sample size, year of publication), participant characteristics (age, sex, Mallampatti grade, and body mass index [BMI]), details on intervention and control groups (e.g., type of VL used), and outcomes (complication rate, first-attempt success rate, total intubation time, and injury site). Any discrepancies in the data extraction process were resolved as previously mentioned. If certain data were absent from the published articles, we contacted the corresponding authors via email to obtain the missing information.

### 2.6. Risk of bias assessment

The quality of RCTs was evaluated using the Cochrane risk of bias tool (RoB 2). This tool assesses bias risk across five domains categorized as low risk, high risk, or having some concerns regarding bias. Two reviewers independently conducted the bias assessment, and any disagreements were addressed through discussion if needed.

### 2.7. Evidence certainty assessment

The reliability of evidence for each outcome was determined using the Grading of Recommendations, Assessment, Development, and Evaluation framework. This method evaluates several criteria, including the risk of bias, inconsistency, indirectness, imprecision, and publication bias, to establish the overall reliability of the evidence. Based on this evaluation, ratings of high, moderate, low, and very low were assigned to each outcome. Two reviewers independently assessed the evidence certainty, and disagreements were resolved through discussion or consultation with a third reviewer as needed.

### 2.8. Statistical analysis

For binary outcomes, risk ratios (RRs) with 95% confidence intervals (CIs) were computed. For continuous data, mean differences (MDs) with 95% CIs were calculated. Using the I^2^ statistic, variability among the studies was quantified. Heterogeneity was categorized as low, moderate, or high based on I^2^ values of 25%, 50%, and 75%, respectively. A random-effects model utilizing the DerSimonian–Laird method was applied irrespective of the level of heterogeneity. If heterogeneity was significant for the primary outcome (i.e., complication rate), subgroup analysis was conducted to explore its sources. Sensitivity analyses were conducted by sequentially excluding one study at a time. The presence of publication bias was assessed visually using funnel plots and statistically using Egger’s test. All statistical calculations were performed using Review Manager (RevMan, version 5.4, The Cochrane Collaboration, Copenhagen, Denmark) and comprehensive meta-analysis. Statistical significance was set at a two-tailed *P*-value < 0.05.

A trial sequential analysis (TSA) was conducted to evaluate the robustness of the evidence and determine if the cumulative data were adequately powered to detect the specified effect sizes, as previously reported [27,28]. The TSA software (version 0.9.5.10 Beta) was used in the analysis, with an alpha of 5%, a power of 80%, and an observed relative risk reduction. If the cumulative Z-curve crossed the TSA monitoring boundary or the required information size, the results were deemed definitive. Otherwise, further studies would be warranted to establish a conclusive outcome.

## 3. Results

### 3.1. Study selection

**Fig 1:**
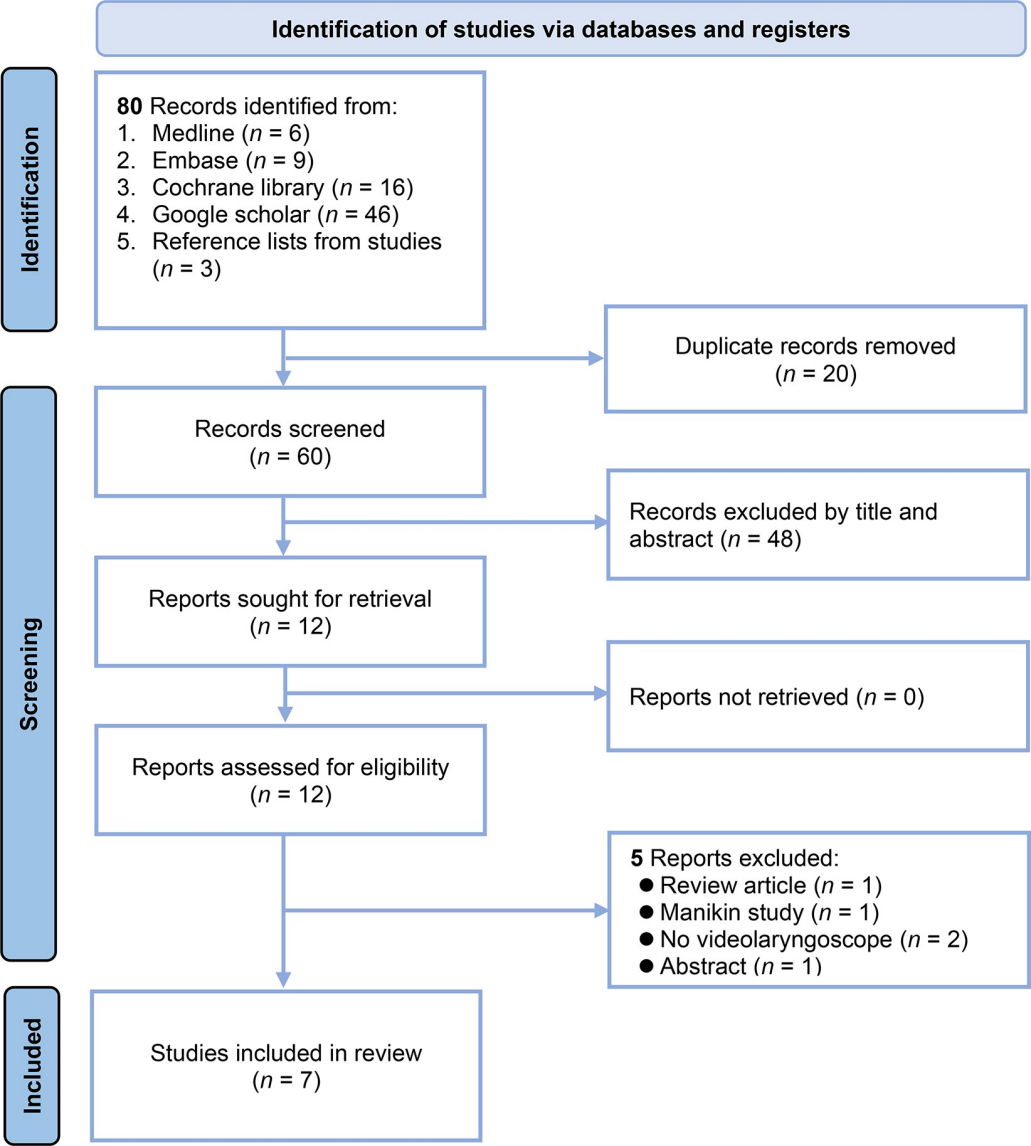
Flowchart of study selection for meta-analysis.

The initial literature search across these databases resulted in a total of 80 records. These databases were Medline (6 records), Embase (9 records), Cochrane Library (16 records), Google Scholar (46 records), and reference lists from other studies (3 records) (Fig 1). After the removal of 20 duplicate records, 60 were screened for relevance based on their titles and abstracts. The screening resulted in the exclusion of 48 records that failed to meet the inclusion criteria or were deemed irrelevant to the research questions. Subsequently, the full texts of the remaining 12 records were sought for more detailed evaluation. All 12 records were retrieved and assessed for eligibility. During this phase, five records were excluded for the following reasons: review article (1 record); involving manikin (1 record); not using videolaryngoscopy, which was a critical inclusion criterion (2 records); and only an abstract (1 record) **(**S2 Table**)**. Ultimately, seven studies that met all the inclusion criteria were identified and thus included in the meta-analysis (S2 Table) [17,24,25,29–32].

### 3.2. Characteristics and quality of studies

This meta-analysis synthesized data from seven RCTs, incorporating 716 participants (Table 2). The clinical settings of the included studies were diverse, with the majority (five studies) focusing on cardiac surgery [17,24,25,29,31]. One study examined an intensive care unit population requiring TEE evaluation [30], whereas another included both cardiac and noncardiac surgical patients [32]. The participants’ ages spanned from 44 to 69 years across studies conducted in India, Japan, Turkey, Spain, Thailand, and China. Their BMI ranged from 22 to 30 kg/m^2^, suggesting that they were generally nonobese. In addition, most participants had a Mallampati score ≤ 2, indicating that airway management was likely straightforward in this population. The trials compared four models of VL—McGrath (three trials) [17,24,25], Medicam (one trial) [29], C-MAC (two trials) [30,31], and Kingtaek (one trial) [32]—with conventional methods, such as blind insertion (five trials) [17,29–32] and Macintosh laryngoscopes (two trials) [24,25]. In the control groups, various techniques were employed to facilitate TEE probe insertion. Three studies [17,29,30] used similar methods, which involved lifting and flexing the mandible by placing the thumb of the left hand into the patient’s mouth. The TEE probe was then gently inserted through the midline into the mouth using the right hand, while maintaining the patient’s head in a neutral position. Another study [31] also employed mandibular subluxation, with the thumb of the left hand placed into the patient’s mouth and the remaining fingers positioned around the submandibular region. During the procedure, the patient’s head was kept in a neutral position to facilitate smooth passage of the probe into the esophagus. Macintosh laryngoscopes were used in the control groups of two studies [24,25] to aid in TEE probe insertion. The study by Yang et al.,[32] used a blind insertion technique but did not provide detailed information on the specific method employed.

**Table 2:** Characteristics of seven randomized controlled studies with a total of 716 patients.

Studies	Population	Age (years)	Male (%)	BMI (kg/m^2^)	Malampatti grade≤2	N	VL	Control	Country
Borde 2022 [29]	Cardiac surgery	55 vs. 54	29 vs. 36.7	24 vs. 24	88.7 vs. 92.1	263	Medicam	Blind technique	India
Ishida 2016 [24]	Cardiac surgery	69 vs. 69	64 vs. 68	23 vs. 22	96 vs. 98	100	McGrath	Macintosh	Japan
Kavrut 2017 [17]	Cardiac surgery	58 vs. 60	57 vs. 54	24 vs. 23	100 vs. 100	83	McGrath	Blind insertion	Turkey
Kimura 2016 [25]	Cardiac surgery	NA	NA	NA	NA	80	McGrath	Macintosh	Japan
Taboada 2024 [30]	ICU population	65 vs. 66	74 vs. 70	29 vs. 30	83.4 vs. 71.7	100	C-MAC	Blind technique	Spain
Vijitpavan 2015 [31]	Cardiac surgery	62 vs. 64	60 vs. 73	23 vs. 24	NA	30	C-MAC	Blind technique	Thailand
Yang 2023 [32]	Mixed surgery†	46 vs. 44	59 vs. 55	23 vs. 23	93 vs. 93⁋	60	Kingtaek	Blind technique	China

VL: Videolaryngoscope

⁋assessed using Cormach-Lehane grade; BMI: Body mass index

†cardiac and non-cardiac surgery; aICU: Intensive care unit.

**Fig 2:**
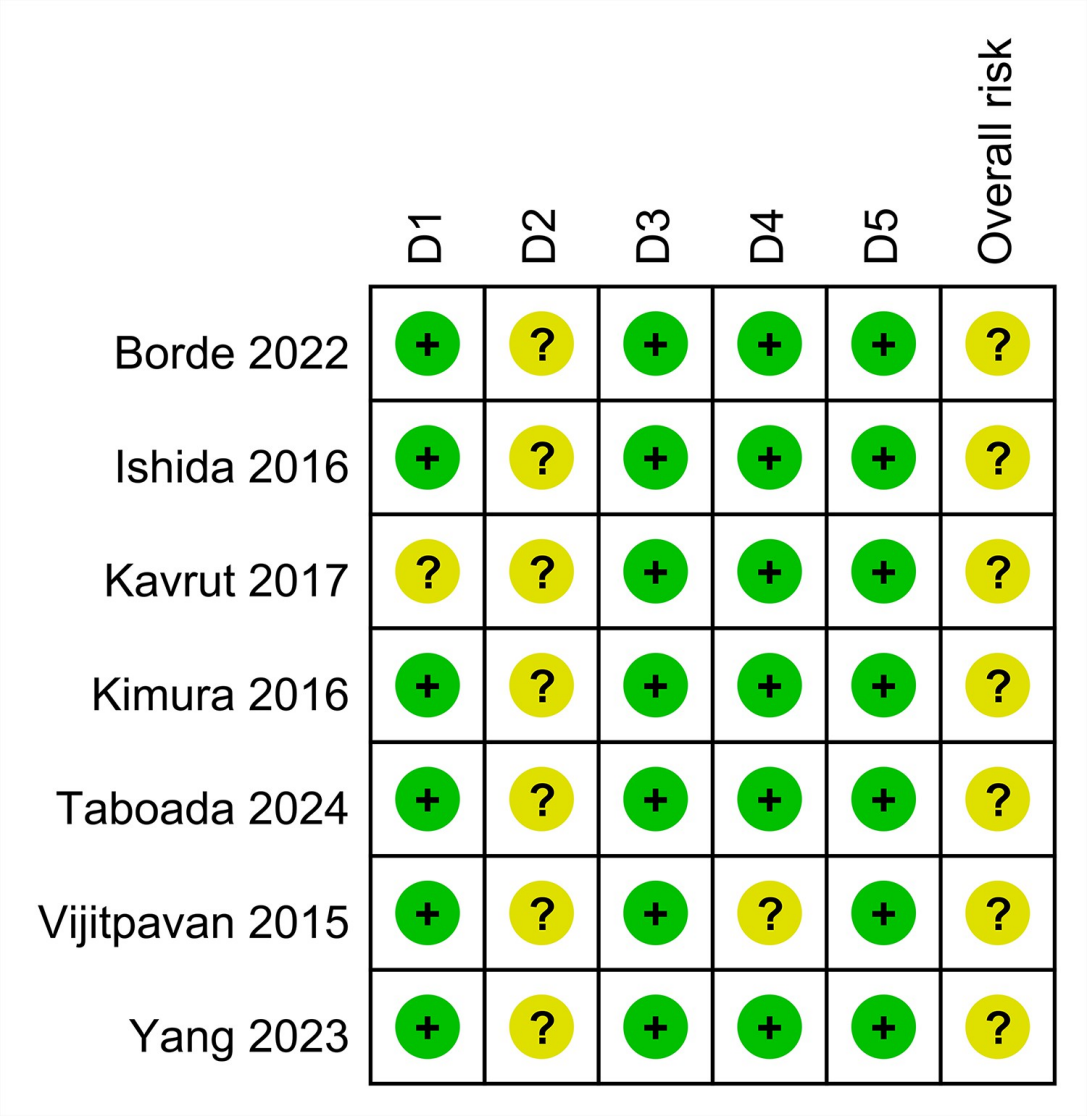
Risk of bias assessment across studies. Domains: D1: Bias arising from the randomization process. D2: Bias due to deviations from intended intervention. D3: Bias due to missing outcome data. D4: Bias in measurement of the outcome. D5: Bias in selection of the reported result.

The quality of the studies is shown in Fig 2 and S3 Table. Due to the lack of detailed information on the randomization process, one study was categorized as having some concern regarding bias in the randomization domain [17]. All studies were evaluated as having some concern regarding bias in the domain of deviations from intended interventions mainly because it is challenging to blind clinicians, which could influence the outcomes. All studies were considered to have some concerns regarding overall risk of bias.

### 3.4. Outcomes

#### 3.4.1. Primary outcome—complication rate

**Fig 3:**
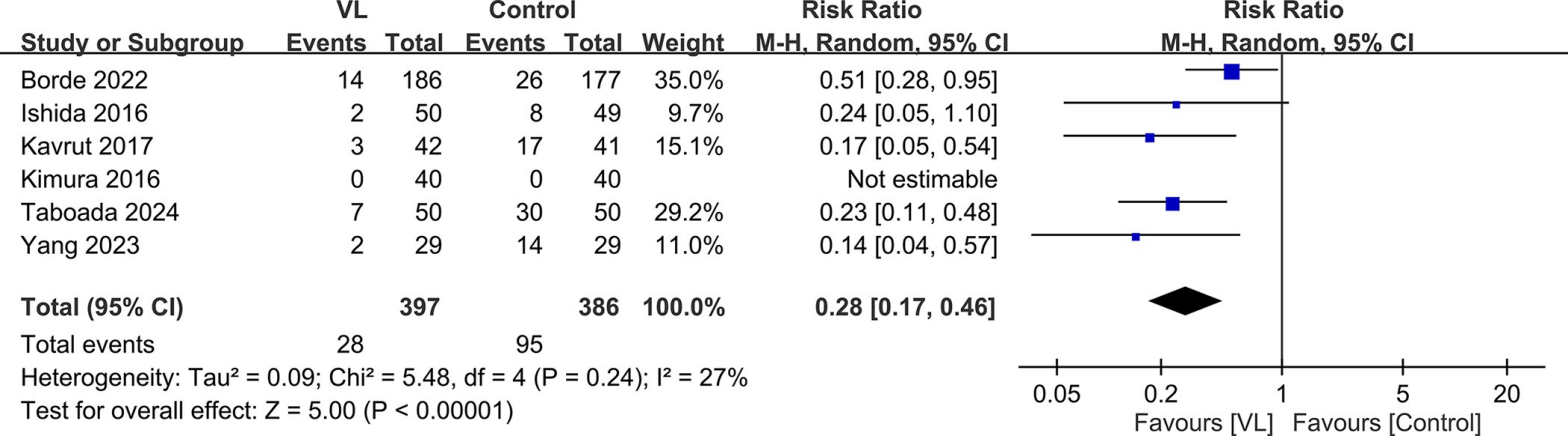
Forest plot showing the complications rates between videolaryngoscope (VL) and control groups. M-H: Mantel-Haenszel, CI: Confidence interval.

Raw data used in current meta-analysis was available in S4 Table. Data from six studies involving 783 participants were obtained to analyze complication rates associated with TEE probe insertion using VL versus control methods [17,24,25,29,30,32]. Overall, the complication rates were notably different, with 7.1% in the VL group compared with 24.6% in the control group. The pooled analysis revealed a significant reduction in the risk of complications with the use of VL (RR: 0.28, 95% CI: 0.17–0.46 < 0.00001) (Fig 3). The heterogeneity among the studies was low (I^2^ = 27%), indicating relatively consistent effect sizes across varied clinical settings and populations. In five studies, the findings were consistent, with RRs ranging from 0.14 to 0.51, all favoring the VL [17,24,29,30,32]. One study reported no complications in either group, thus precluding an RR estimation for that particular study [25].

**Fig 4:**
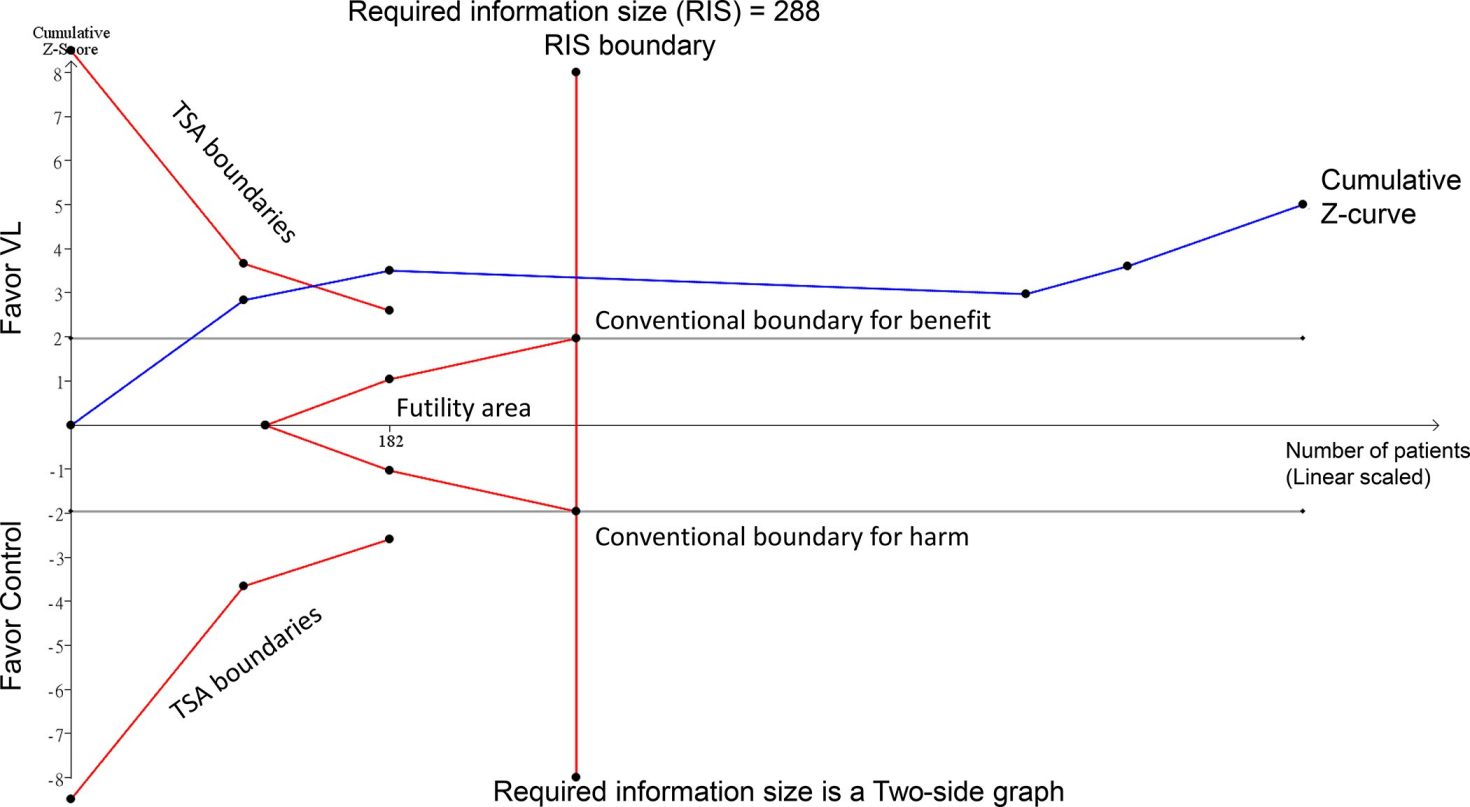
Trial sequential analysis (TSA) for complication rate.

The TSA was conducted to determine the required information size (RIS) and assess the reliability of the cumulative evidence regarding complication rates from the use of VL versus control techniques. The TSA indicated that the cumulative Z-curve crossed the RIS, providing strong evidence of a significant reduction in complication rates with the use of VL (Fig 4).

**Fig 5:**
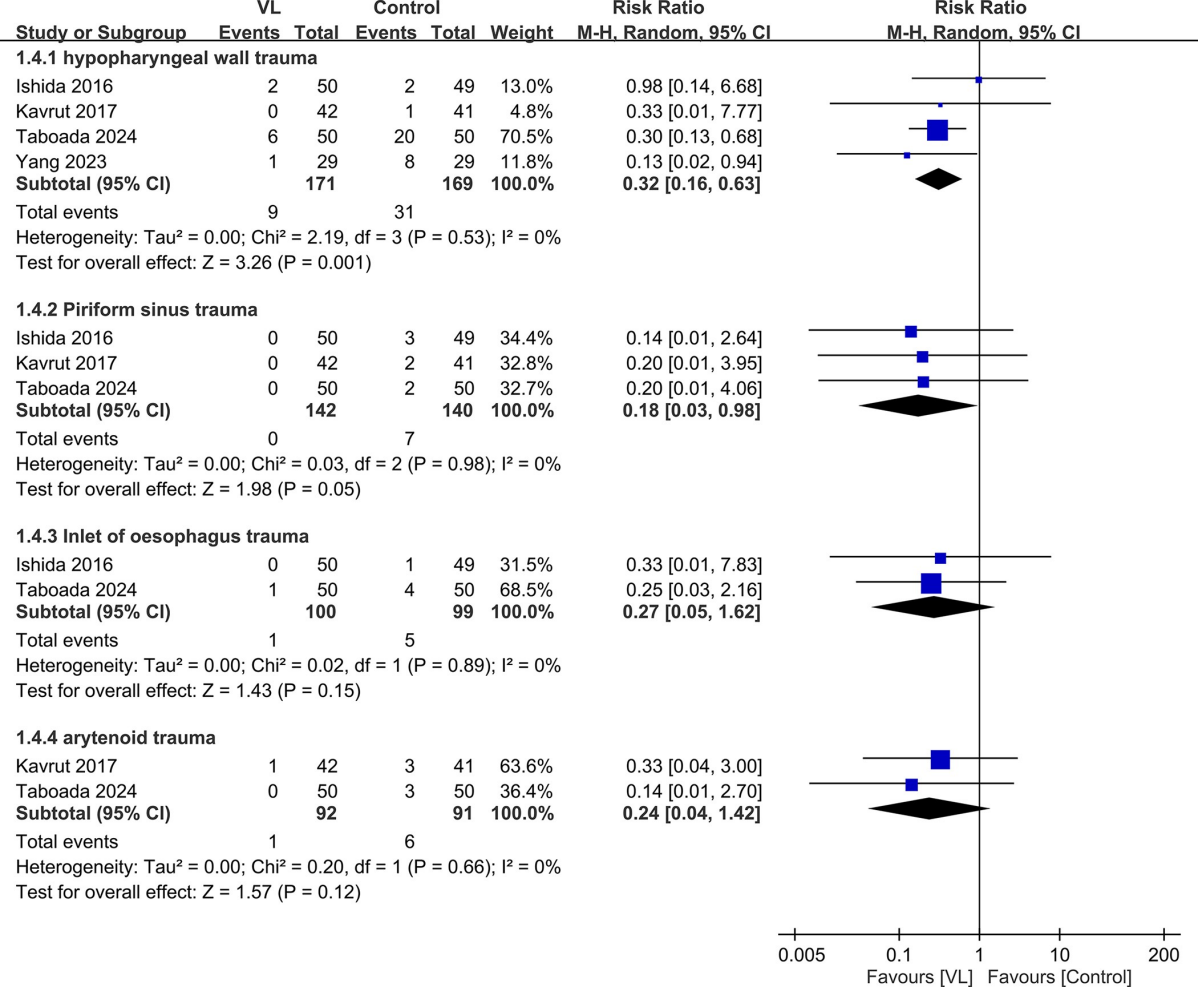
Forest plot showing the sites of injury between videolaryngoscope (VL) and control groups. M-H: Mantel-Haenszel, CI: Confidence interval.

Only a few studies provided details on the specific locations of TEE-associated injuries. The meta-analysis revealed that VL significantly reduced the risk of trauma to the hypopharyngeal wall (RR: 0.32, 95% CI: 0.16–0.63, *P* = 0.001, I^2^ = 0%). However, no significant benefits were observed in other areas such as the pyriform sinus (RR: 0.18, 95% CI: 0.03–0.98, *P* = 0.05, I^2^ = 0%), esophageal inlet (RR: 0.27, 95% CI: 0.05–1.62, *P* = 0.15, I^2^ = 0%), and arytenoids (RR: 0.24, 95% CI: 0.04–1.42, *P* = 0.12, *I*^*2*^ = 0%) (Fig 5).

#### 3.4.2. Secondary outcome—first-attempt success rate

**Fig 6:**
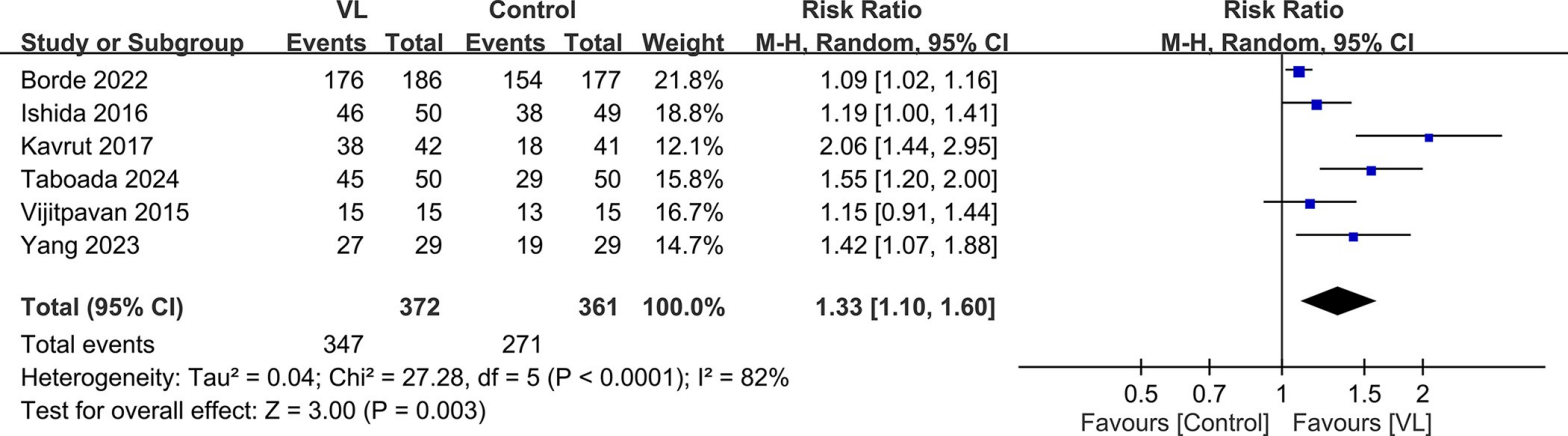
Forest plot showing the first attempt success rate between videolaryngoscope (VL) and control groups. M-H: Mantel-Haenszel, CI: Confidence interval.

The first-attempt success rates were 93.3% and 75.1% in the VL and control groups, respectively. The results of the pooled analysis, involving a total of 733 participants, indicated that VL significantly increased the likelihood of successful probe insertion on the first attempt by 33% compared with conventional methods (RR: 1.33, 95% CI: 1.10–1.60, *P* = 0.003) (Fig 6) [17,24,29–32]. Although the individual study results indicated a consistent trend toward better performance with VL, the heterogeneity was significant. The range of effect sizes varied from 1.09–2.06, with the highest RR observed in the Kavrut 2017 study [17], in which all patients had a Mallampati score ≤ 2, suggesting easier airway access.

**Fig 7:**
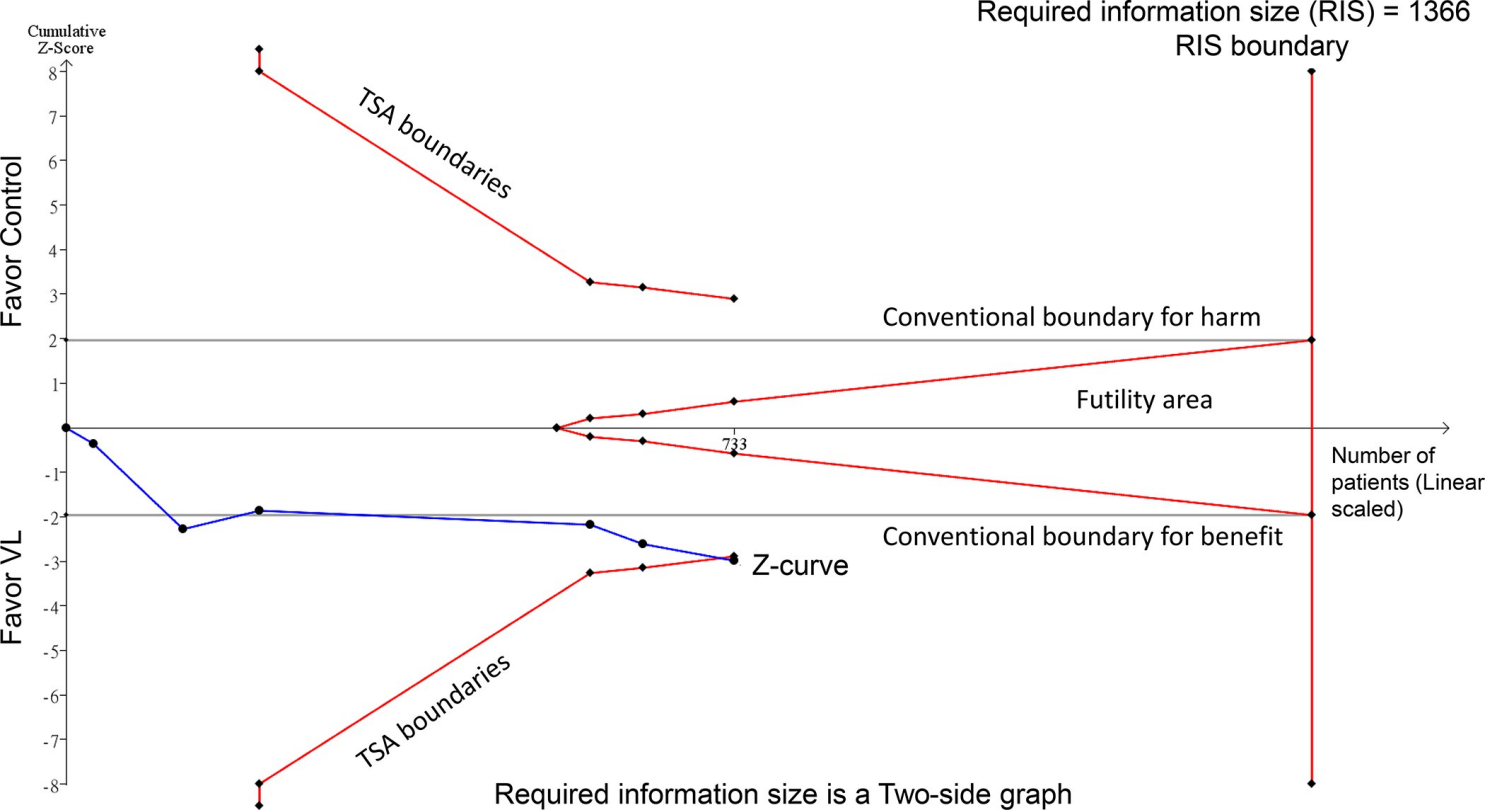
Trial sequential analysis (TSA) for first attempt success rate.

The TSA revealed that although the RIS was not reached, the cumulative Z-curve crossed the trial sequential monitoring boundary. This crossing indicates robust evidence supporting a significant increase in the first-attempt success rate when using VL (Fig 7).

#### 3.4.3. Secondary outcome—TEE insertion time

**Fig 8:**
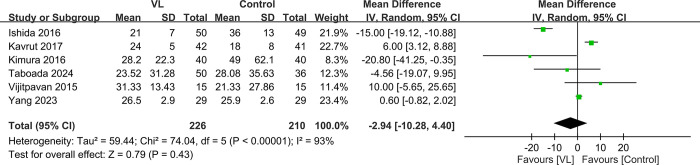
Forest plot showing the insertion time between videolaryngoscope (VL) and control groups. IV: Invariance, CI: Confidence interval.

**Fig 9:**
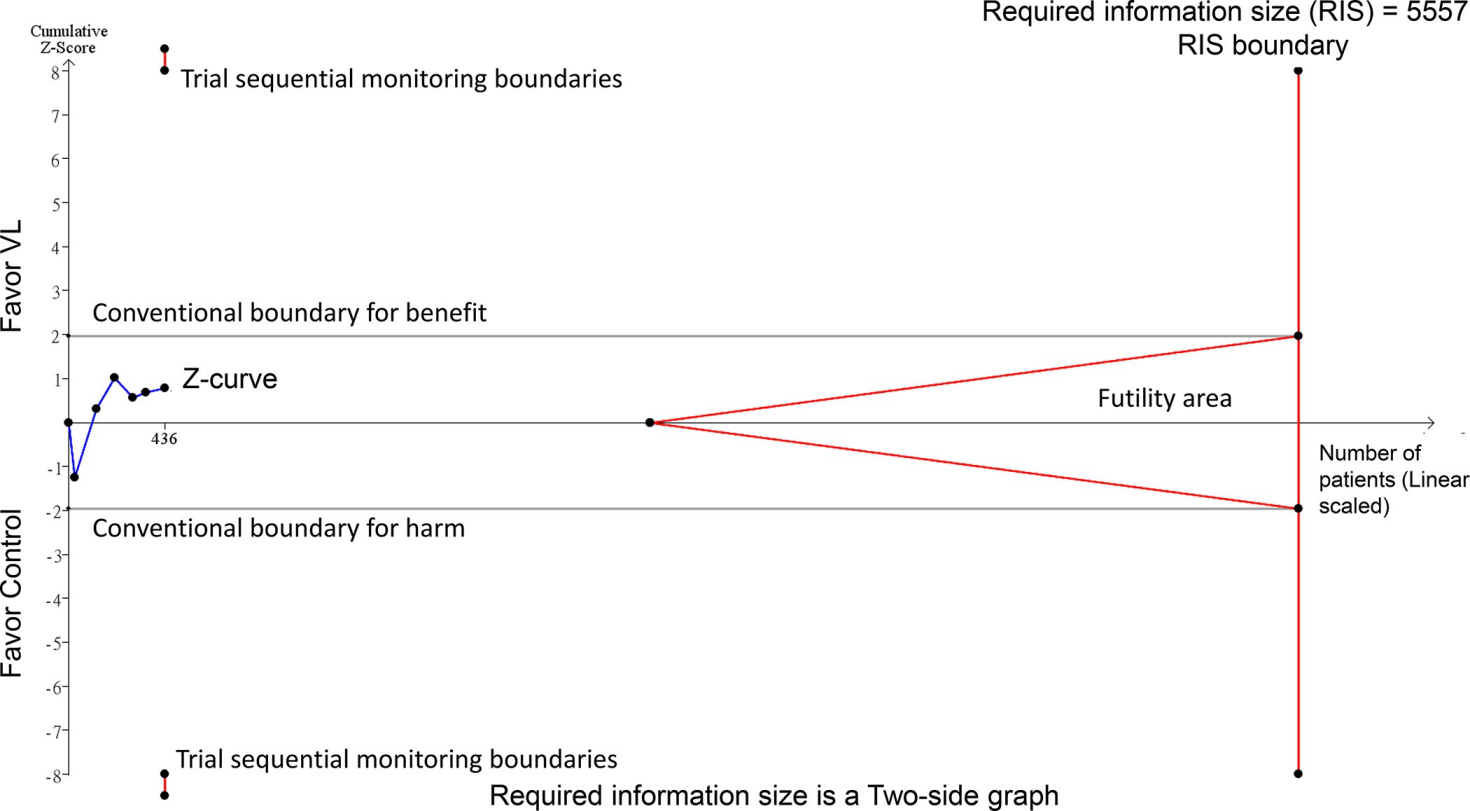
Trial sequential analysis (TSA) for insertion time.

The meta-analysis of six studies revealed no significant difference in the TEE insertion time between the VL and control groups, with an MD of −2.94 s (95% CI: −10.28–4.4, *P* = 0.43) (Fig 8) [17,24,25,30–32]. The high heterogeneity observed indicated that this outcome could be susceptible to variations in clinical settings. Despite the effect size ranging from −20.8 to 10 s across studies, such variations in time appeared to be clinically insignificant. The TSA revealed that the Z-curve did not cross into the futility area, suggesting that the evidence was not conclusive (Fig 9).

#### 3.4.4. Publication bias

**Fig 10:**
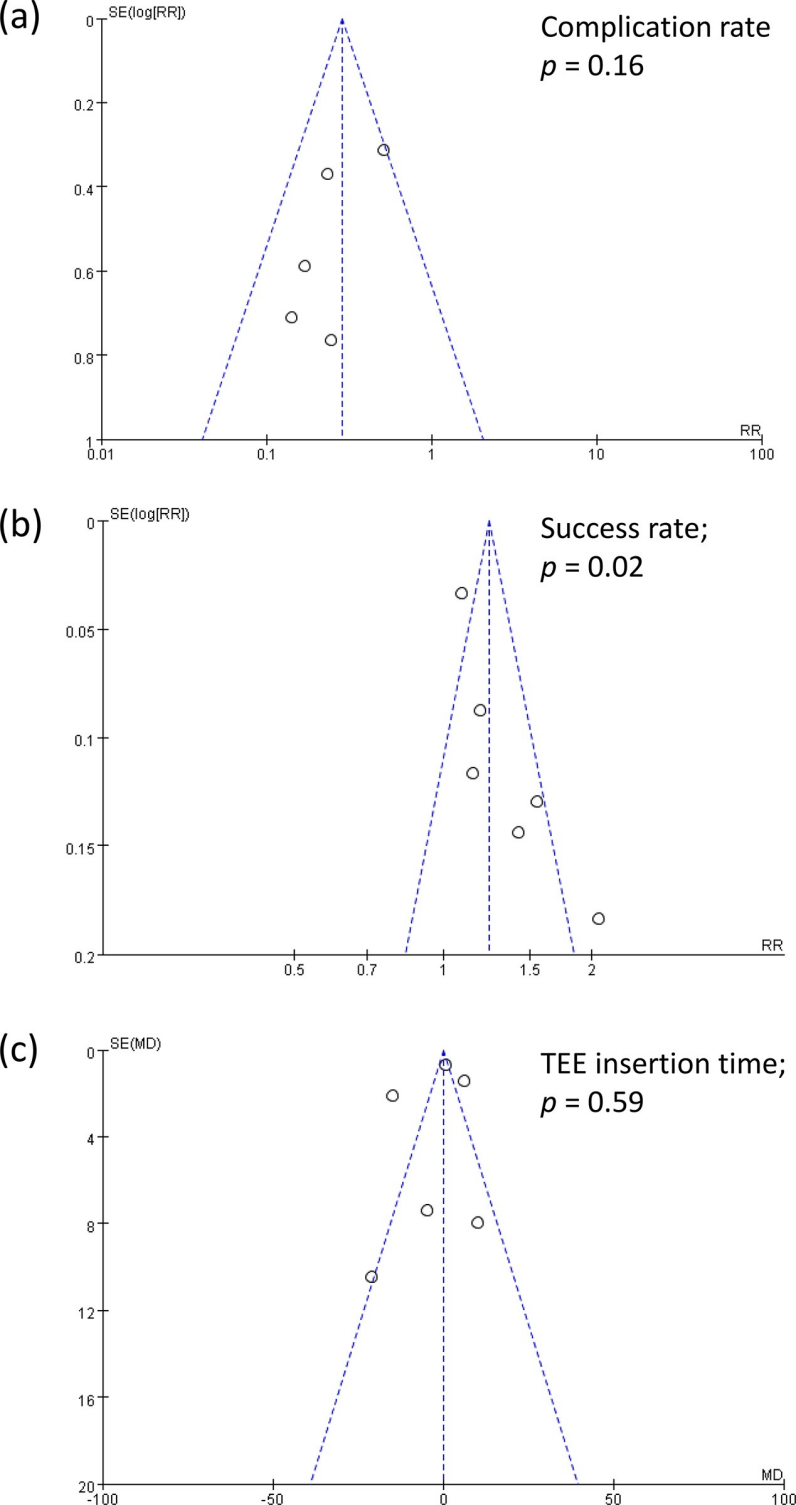
The figure presents three funnel plots evaluating publication bias in studies on different outcomes. Panel (a) complication rates; Panel (b) first attempt success rates; Panel (c) transesophageal echocardiography (TEE) insertion time.

Egger’s test revealed no publication bias for the complication rate (*P* = 0.16) (Fig 10A) and TEE insertion time (*P* = 0.59) (Fig 10C). However, it suggested potential publication bias for the first-attempt success rate (*P* = 0.02) (Fig 10B).

#### 3.4.5. Sensitivity analysis

Sensitivity analysis, conducted by excluding one study at a time, yielded consistent results regarding complication rate, first-attempt success rate, and TEE insertion time.

#### 3.4.6. Certainty of evidence

Table 3 shows the summary of findings and the certainty of evidence, which was moderate for the complication rate, very low for the first-attempt success rate, and low for the TEE insertion duration.

**Table 3:** Summary of outcomes and certainty of evidence based on the Grading of Recommendations Assessment, Development and Evaluation (GRADE) approach.

Outcomes	n†	Participants	Certainty assessment (Domains)					Effect size [95% CI]; p-value	I^2^	Sensitivity analysis	Certainty
			A	B	C	D	E				
Complication rate	6	783	S	NS	NS	NS	NS	RR 0.28 [0.17, 0.46]; *p* < 0.00001	27%	Consistent	⨁⨁⨁◯ Moderate
First attempt success rate	6	733	S	S	NS	NS	S	RR 1.33 [1.10, 1.60]; *p* = 0.003	82%	Consistent	⨁◯◯◯ Very low
TEE insertion time	6	436	S	S	NS	NS	NS	MD -2.94 [-10.28, 4.40]; *p* = 0.43	93%	Consistent	⨁⨁◯◯ Low

A: Risk of bias; B: Inconsistency; C: Indirectness; D: Imprecision; E: Publication bias; NS: Not serious;S: Serious.

RR: Risk Ratio; CI: Confidence Interval; †number of studies or datasets; TEE: Transesophageal echocardiography probe.

## 4. Discussion

Our meta-analysis of 7 RCTs involving 716 participants revealed that the use of VL significantly reduced the complication rate (RR: 0.28) and increased the first-attempt success rate (RR: 1.33) compared with conventional methods for TEE probe insertion. These findings were confirmed by TSA. However, no significant difference was observed in the TEE insertion time between the two groups (MD: −2.94 s). The certainty of evidence was moderate for the complication rate, very low for the first-attempt success rate, and low for the TEE insertion time. VL significantly reduced the risk of trauma to the hypopharyngeal wall but showed no significant benefits in other areas, such as the pyriform sinus, esophageal inlet, and arytenoids. The sensitivity analysis yielded consistent results, and no publication bias was detected for the complication rate and TEE insertion time, although potential bias was observed for the first-attempt success rate.

A retrospective study of 7,200 intraoperative examinations in cardiac surgical patients reported incidences of TEE-related upper gastrointestinal bleeding and perforation at 0.03% and 0.01%, respectively [10]. Similarly, another study reported major gastrointestinal complication rate and mortality rate of 0.09% and 0.02%, respectively [33]. A more recent multicenter prospective audit involving 22,314 intraoperative TEE examinations reported morbidity and mortality rates of 0.08% and 0.03%, respectively [34]. As that these incidences are mainly derived from retrospective analyses or audits, the actual frequency of TEE-associated complications might be significantly underreported. In the current meta-analysis, the complication rate was as high as 24.6% in the control group, suggesting that TEE-associated complications are more common than previously thought. Considering the widespread use of TEE in surgical, intensive care, and emergency settings, many patients may experience these complications. The consensus guidelines from the American Society of Echocardiography and the Society of Cardiovascular Anesthesiologists highlight the importance of minimizing multiple attempts of TEE probe insertion in patients under anesthesia [35]. Strategies that effectively minimize such attempts are vital and should be integrated into clinical protocols.

Our finding indicated that the use of VL can significantly enhance patient safety during TEE probe insertion. The lower complication rate observed with VL could be attributed to several factors. First, VL provides a clear, magnified view of the laryngeal structures, enabling a more precise manipulation of the TEE probe and reducing the risk of trauma to the surrounding tissues. Second, the use of VL may facilitate easier and more rapid identification of the esophageal inlet, which reduces the need for multiple attempts and minimizes the contact duration between the probe and the oropharyngeal structures. The TSA confirmed that the cumulative evidence supports the substantial reduction in complication rates with VL use. This finding strengthens the validity of the results and indicates that further trials may not be necessary to confirm the benefit of VL in reducing complications. Subgroup analysis based on the injury locations revealed that VL significantly reduced the risk of trauma to the hypopharyngeal wall. This finding is clinically relevant as injuries to the hypopharyngeal wall can lead to serious complications, such as perforation and mediastinitis [36]. However, no significant benefits were observed in other areas, such as the pyriform sinus, esophageal inlet, and arytenoids. The small number of studies reporting specific injury sites may have contributed to the lack of significant differences in these subgroups.

Our meta-analysis builds upon and expands the findings of the previous meta-analysis conducted by Namekawa et al. [26] who also investigated the utility of videolaryngoscopy for TEE probe insertion. While our study and the meta-analysis by Namekawa et al. [26] demonstrated the benefits of videolaryngoscopy in reducing complications and the number of attempts required for a successful probe insertion, our meta-analysis involved a larger number of studies and participants, providing a more comprehensive and robust assessment of the available evidence. The meta-analysis conducted by Namekawa et al. [26] included 3 RCTs involving 266 participants, whereas our meta-analysis incorporated seven trials involving 716 participants. The large sample size in our study enabled more precise effect size estimates and enhanced the generalizability of the findings. Furthermore, the inclusion of a larger number of studies enabled us to conduct subgroup analyses based on the location of injuries, which were not reported in the previous meta-analysis [26]. Another key difference between the two meta-analyses is the patient population. Namekawa et al.’s [26] meta-analysis focused only on patients undergoing TEE in the operating room setting, whereas our meta-analysis included studies involving patients outside the operating room, e.g., intensive care units. Our data enables a more comprehensive assessment of the utility of videolaryngoscopy in various clinical settings.

The first-attempt success rate is a crucial outcome in the evaluation of TEE probe insertion techniques as it reflects the ease and efficiency of the procedure. Our meta-analysis revealed that the use of VL significantly increases the likelihood of successful probe insertion on the first attempt compared with conventional methods (RR: 1.33). This finding suggests that VL can enhance the efficiency of TEE probe insertion and reduce the need for multiple attempts, which may be associated with increased patient discomfort and a higher risk of complications. The magnified, high-resolution view of the laryngeal structures provided by the VL enables a more precise manipulation of the TEE probe, allowing operators to navigate the anatomical landmarks more easily and identify the optimal path for insertion. However, it is important to acknowledge the significant heterogeneity observed among the studies included in the analysis (I^2^ = 82%). Such a heterogeneity could be attributed to variations in operator experience, patient characteristics, and the specific type of VL used. The subgroup analysis based on the VL type was limited by the small number of studies using each device. Operator experience is another critical factor that may influence the first-attempt success rate. In the included studies, the TEE probe insertion was performed by experienced operators, which may have contributed to the high success rates in both the VL and control groups. The impact of VL on the learning curve and skill acquisition of novice operators remains an important area for future research as the enhanced visualization provided by this technique may facilitate training and improve success rates among practitioners with less experience.

A small-scale study involving 153 adult patients undergoing cardiac surgery identified male sex, obesity, and high Mallampati and Cormack–Lehane scores as predictors of difficult TEE probe insertion in anesthetized patients [16]. As these factors are also associated with difficult intubation, similar mechanisms may underlie challenges with TEE probe insertion. For instance, the modified Mallampati classification, which evaluates the visibility of the base of the uvula and the oropharyngeal walls, is a significant predictor of difficult intubation [37]. High modified Mallampati scores indicate potential airway obstruction by the tongue. Consequently, it is understandable that these higher scores are associated with more challenging TEE probe insertion. Similarly, another study reported that the modified Mallampati classification is a significant factor in determining the success of TEE probe insertion in nonsedated patients [38]. The recognition of these risk factors could enable clinicians to foresee potential difficulties with TEE probe insertion and implement preventive measures, such as employing VL, to reduce the risk of complications. Further research may be warranted to explore other potential risk factors [39] for difficult intubation and TEE probe insertion. Such studies could provide a broader understanding of how various anatomical characteristics impact the ease of probe insertion, potentially leading to improved protocols and outcomes in cardiac surgery and critical care settings.

This meta-analysis has several limitations that need to be considered when interpreting the results. First, the included studies had relatively small sample sizes, which may limit the power to detect significant differences in some outcomes, particularly the TEE insertion time. Second, a significant heterogeneity was detected among the studies for the first-attempt success rate and TEE insertion time, which could be attributed to variations in clinical settings, operator experience, and VL types used. Third, the majority of the participants had a Mallampati score ≤ 2, suggesting that the findings may not be generalizable to patients with more difficult airways. Fourth, blinding of the operators was impossible due to the nature of the intervention, which may have introduced performance bias. Fifth, most patients included in the analyzed studies had a relatively normal BMI, ranging from 22 to 30 kg/m^2^. This limitation may affect the generalizability of our findings to patients with higher BMIs. Future studies should include a more diverse range of patient BMIs to better understand the impact of videolaryngoscopy on TEE probe insertion outcomes in these populations. In addition, critically ill patients, particularly those with prolonged hospital stays or those receiving mechanical ventilation, may have increased tissue fragility due to factors such as malnutrition, edema, or the use of corticosteroids. Given that only one study focused on the ICU population, a limited number of studies have constrained our ability to evaluate the impact of this factor on the outcomes of interest. Lastly, adequate oral opening is required for a successful VL insertion. In cases where oral opening is limited, blind insertion may be preferable, but the advantages of VL may be diminished.

In conclusion, this meta-analysis provides evidence that the use of VL for TEE probe insertion is associated with a significantly lower complication rate and higher first-attempt success rate than conventional methods. These findings indicate that VL may enhance patient safety and improve the efficiency of TEE probe insertion. The certainty of evidence was moderate for the complication rate and very low for the first-attempt success rate, emphasizing the need for further high-quality RCTs to strengthen the evidence base. Future studies should also focus on standardizing the definition of complications and investigate the impact of operator experience and use of different types of VL on the outcomes.

## Supporting information

S1 Table PRISMA checklist. (DOCX)

S2 Table Studies included and excluded. (DOCX)

S3 Table Risk of bias for each study. (DOCX)

S4 Table Raw data used in current meta-analysis. (DOCX)
