# Supplementary material for: Efficacy and safety of videolaryngoscopes for transesophageal echocardiography probe insertion: A trial sequential meta-analysis
Source: PLoS One. 2024 Oct 10;19(10):e0311234. doi: 10.1371/journal.pone.0311234 (PMC11466393; doi:10.1371/journal.pone.0311234)
Supplement: S2 Table — (DOCX) [file pone.0311234.s002.docx]

**S2 Table.** Studies included and excluded

| **Studies included** | |
| --- | --- |
| 1. Borde, D., C, K., Jasapara, A., Shetty, V., Juvekar, N., Desurkar, V., . . . Koshy, T. (2022). Use of a Video Laryngoscope to Reduce Complications of Transesophageal Echocardiography Probe Insertion: A Multicenter Randomized Study. Journal of Cardiothoracic & Vascular Anesthesia, 36(12), 4289-4295. doi:https://dx.doi.org/10.1053/j.jvca.2022.07.017 2. Ishida, T., Kiuchi, C., Sekiguchi, T., Tsujimoto, T., & Kawamata, M. (2016). McGRATH MAC video laryngoscope for insertion of a transoesophageal echocardiography probe. European Journal of Anaesthesiology, 33(4), 263-268. doi:https://dx.doi.org/10.1097/EJA.0000000000000367 3. Kavrut Ozturk, N., & Kavakli, A. S. (2017). Use of McGrath MAC Videolaryngoscope to Assist Transesophageal Echocardiography Probe Insertion in Intubated Patients. Journal of Cardiothoracic & Vascular Anesthesia, 31(1), 191-196. doi:https://dx.doi.org/10.1053/j.jvca.2016.03.150 4. Kimura, T., Katoh, T., Ogasawara, T., Mimuro, S., Makino, H., Suzuki, A., & Sato, S. (2016). The McGRATH MAC video laryngoscope facilitates probe insertion during transesophageal echocardiography. [Japanese]. Japanese Journal of Anesthesiology, 65(1), 68-74. 5. Taboada, M., Carinena, A., Estany-Gestal, A., Iglesias-Alvarez, D., Veiras, S., Martinez, A., . . . Seoane-Pillado, T. (2024). Videolaryngoscope versus conventional technique for insertion of a transesophageal echocardiography probe in intubated ICU patients (VIDLARECO trial): A randomized clinical trial. Anaesthesia Critical Care & Pain Medicine, 43(2), 101346. doi:https://dx.doi.org/10.1016/j.accpm.2024.101346 6. Vijitpavan, A., Saelao, A., Purintrapiban, B., Kingsakul, W., & Pootongngen, W. (2015). Comparison of C-MAC D-Blade videolaryngoscope with conventional blind technique for transesophageal echocardiography probe insertion. Thai Journal of Anesthesiology, 41(3), 179-188. 7. Yang, W., Zhou, L., Bi, J., Wang, P., & Huang, Q. (2023). Application of video laryngoscope assisted transesophageal echocardiography probe insertion in acute severe patients. Guangzhou Medical Journal, 54(3), 62-66. | |
| **Reasons** | **Studies excluded** |
| Review article | Namekawa, M., Tsujimoto, Y., Banno, M., Kataoka, Y., Tsujimoto, H., Inaba, Y., & Fujiwara, T. (2020). Videolaryngoscopy for transesophageal echocardiography probe insertion: a systematic review and meta-analysis of randomized controlled trials. Journal of Anesthesia, 34, 453-463. |
| Manikin study | Kumamoto, T., Tashima, K., Hiraoka, C., Ikuta, Y., & Yamamoto, T. (2021). McGRATH MAC video laryngoscope assistance during transesophageal echocardiography may reduce the risk of complications: a manikin study. BMC Anesthesiology, 21, 1-6. |
| No videolaryngoscope | 1. Na, S., Kim, C. S., Kim, J. Y., Cho, J. S., & Kim, K. J. (2009). Rigid laryngoscope-assisted insertion of transesophageal echocardiography probe reduces oropharyngeal mucosal injury in anesthetized patients. The Journal of the American Society of Anesthesiologists, 110(1), 38-40. 2. Kiran, M., Gadhinglajkar, S., Sreedhar, R., Sukesan, S., Pillai, V., & Panicker, V. (2023). Factors predicting difficulty in insertion of real-time-three-dimensional transesophageal echocardiography probe in adult patients undergoing cardiac surgery. Annals of Cardiac Anaesthesia, 26(1), 12-16. doi:10.4103/aca.aca_287_20 |
| Abstract | Kimura, T., Katoh, T., Mimuro, S., Makino, H., & Sato, S. (2014). The McGRATH MAC is useful when inserting a probe for transesophageal echocardiography. European Journal of Anaesthesiology, 52), 62. |
