# Supplementary material for: Efficacy and safety of videolaryngoscopes for transesophageal echocardiography probe insertion: A trial sequential meta-analysis
Source: PLoS One. 2024 Oct 10;19(10):e0311234. doi: 10.1371/journal.pone.0311234 (PMC11466393; doi:10.1371/journal.pone.0311234)
Supplement: S3 Table — (DOCX) [file pone.0311234.s003.docx]

**S3 table.** Risk of bias for each study

| **Study ID** | **D1** | **D2** | **D3** | **D4** | **D5** | **Overall risk of bias** |
| --- | --- | --- | --- | --- | --- | --- |
| Borde 2022 | **Low** | **Some concern** | **Low** | **Low** | **Low** | **Some concern** |
| Ishida 2016 | **Low** | **Some concern** | **Low** | **Low** | **Low** | **Some concern** |
| Kavrut 2017 | **Some concern** | **Some concern** | **Low** | **Low** | **Low** | **Some concern** |
| Kimura 2016 | **Low** | **Some concern** | **Low** | **Low** | **Low** | **Some concern** |
| Taboada 2024 | **Low** | **Some concern** | **Low** | **Low** | **Low** | **Some concern** |
| Vijitpavan 2015 | **Low** | **Some concern** | **Low** | **Some concern** | **Low** | **Some concern** |
| Yang 2023 | **Low** | **Some concern** | **Low** | **Low** | **Low** | **Some concern** |

D1:Bias arising from the randomization process

D2: Bias due to deviations from the intended interventions

D3: Bias due to missing outcome data

D4: Bias in measurement of the outcome

D5: Bias in selection of the reported results

**S4 Table.** Raw data used in current meta-analysis (**Name of data extractors**: I-Wen Chen and Wei-Ting Wang; **Date of data extraction**: June 18, 2024)

|  | Outcome |  | VL group | | control group | |  |
| --- | --- | --- | --- | --- | --- | --- | --- |
|  |  |  | events | total | events | total |  |
| Borde 2022 | injury |  | 14 | 186 | 26 | 177 |  |
| Ishida 2016 | injury |  | 2 | 50 | 8 | 49 |  |
| Kavrut 2017 | injury |  | 3 | 42 | 17 | 41 |  |
| Kimura 2016 | injury |  | 0 | 40 | 0 | 40 |  |
| Taboada 2024 | injury |  | 7 | 50 | 30 | 50 |  |
| Wenjie 2023 | injury |  | 2 | 29 | 14 | 29 |  |
|  |  |  |  |  |  |  |  |
|  |  | VL group | | | control group | | |
|  |  | mean | sd | n | mean | sd | n |
| Ishida 2016 | insertion time | 21 | 7 | 50 | 36 | 13 | 49 |
| Kavrut 2017 | insertion time | 24 | 5 | 42 | 18 | 8 | 41 |
| Kimura 2016 | insertion time | 28.2 | 22.3 | 40 | 49 | 62.1 | 40 |
| Taboada 2024 | insertion time | 23.52 | 31.28 | 50 | 28.08 | 35.63 | 36 |
| Vijitpavan 2015 | insertion time | 31.33 | 13.43 | 15 | 21.33 | 27.86 | 15 |
| Wenjie 2023 | insertion time | 26.5 | 2.9 | 29 | 25.9 | 2.6 | 29 |
|  |  |  |  |  |  |  |  |
|  |  |  |  |  |  |  |  |
|  |  |  | VL group | | control group | |  |
|  |  |  | events | total | events | total |  |
| Borde 2022 | first attempt success |  | 176 | 186 | 154 | 177 |  |
| Ishida 2016 | first attempt success |  | 46 | 50 | 38 | 49 |  |
| Kavrut 2017 | first attempt success |  | 38 | 42 | 18 | 41 |  |
| Taboada 2024 | first attempt success |  | 45 | 50 | 29 | 50 |  |
| Vijitpavan 2015 | first attempt success |  | 15 | 15 | 13 | 15 |  |
| Wenjie 2023 | first attempt success |  | 27 | 29 | 19 | 29 |  |
|  |  |  |  |  |  |  |  |
|  |  |  |  |  |  |  |  |
|  |  |  |  |  |  |  |  |
|  |  |  | VL group | | control group | |  |
|  |  |  | events | total | events | total |  |
| Ishida 2016 | Posterior hypopharyngeal wall |  | 2 | 50 | 2 | 49 |  |
| Kavrut 2017 | Posterior hypopharyngeal wall |  | 0 | 42 | 1 | 41 |  |
| Taboada 2024 | Posterior hypopharyngeal wall |  | 6 | 50 | 20 | 50 |  |
| Wenjie 2023 | Posterior hypopharyngeal wall |  | 1 | 29 | 8 | 29 |  |
|  |  |  |  |  |  |  |  |
|  |  |  | VL group | | control group | |  |
|  |  |  | events | total | events | total |  |
| Ishida 2016 | Piriform sinus |  | 0 | 50 | 3 | 49 |  |
| Kavrut 2017 | Piriform sinus |  | 0 | 42 | 2 | 41 |  |
| Taboada 2024 | Piriform sinus |  | 0 | 50 | 2 | 50 |  |
|  |  |  |  |  |  |  |  |
|  |  |  | VL group | | control groiup | |  |
|  |  |  | events | total | events | total |  |
| Ishida 2016 | Inlet of oesophagus |  | 0 | 50 | 1 | 49 |  |
| Taboada 2024 | Inlet of oesophagus |  | 1 | 50 | 4 | 50 |  |
|  |  |  |  |  |  |  |  |
|  |  |  | VL group | | control group | |  |
|  |  |  | events | total | events | total |  |
| Kavrut 2017 | arytenoid |  | 1 | 42 | 3 | 41 |  |
| Taboada 2024 | arytenoids |  | 0 | 50 | 3 | 50 |  |
